# Integrating clinical and economic outcomes in the elderly myeloma management: a comparative cost-effectiveness analysis

**DOI:** 10.3389/fonc.2026.1774263

**Published:** 2026-08-24

**Authors:** Antonio G. Solimando, Vanessa Desantis, Lucia Di Marzo, Giulia de Martino, Simona D’Amore, Leonardo Girardi, Giuseppe Dicuonzo, Alessandro Andriano, Franco Dammacco, Angelo Vacca, Massimo Bilancia, Roberto Ria

**Affiliations:** 1 Department of Precision and Regenerative Medicine and Ionian Area (DiMePRe-J), Unit of Internal Medicine ‘Guido Baccelli’, University of Bari Aldo Moro Medical School, Bari, Italy; 2 Department of Precision and Regenerative Medicine and Ionian Area (DiMePRe-J), Section of Pharmacology, University of Bari Aldo Moro Medical School, Bari, Italy; 3 Allergology and Clinical Immunology Medical Specialty School, University of Bari Aldo Moro Medical School, Bari, Italy; 4 Department of Precision and Regenerative Medicine and Ionian Area (DiMePRe-J), CITEL Telemedicine Research Center, University of Bari Aldo Moro, Bari, Italy

**Keywords:** combination therapies, cost-effectiveness analysis, incremental cost-effectiveness ratio, Markov-cohort models, multiple myeloma

## Abstract

**Introduction:**

Multiple myeloma (MM) is a hematologic malignancy that predominantly affects older adults and poses substantial therapeutic challenges due to the high prevalence of comorbidities and limited treatment tolerability. This study evaluates the cost-effectiveness of four first-line treatment regimens for elderly MM patients, ineligible for autologous stem cell transplantation (ASCT): bortezomib, melphalan, and prednisone (VMP); daratumumab in combination with VMP (D-VMP); lenalidomide and dexamethasone (Rd); and daratumumab combined with Rd (DARA-Rd).

**Methods:**

A state-transition Markov model was developed, with transition probabilities parameterized using data derived from the pivotal ALCYONE and MAIA clinical trials. Cost-effectiveness was assessed from the perspective of the Italian National Health Service using a state-transition model comparing VMP, D-VMP, Rd and DARA-Rd over a 5.25-year horizon. Probabilistic sensitivity analyses (PSA) were conducted to account for parameter uncertainty and summarized through cost-effectiveness acceptability curves (CEACs).

**Results:**

Rd generated 1.53 QALYs for €60,483, whereas D-VMP generated 1.70 QALYs at €88,331, resulting in an ICER of €160,804/QALY. DARA-Rd and VMP were dominated.

**Conclusion:**

The findings indicate that Rd is the strategy with the highest probability of being cost-effective at willingness-to-pay (WTP) thresholds below €250,000 per quality-adjusted life year (QALY) gained. Conversely, for WTP thresholds exceeding €250,000 per QALY gained, D-VMP emerges as the preferred option, offering the most favorable balance between additional costs and health benefits.

## 1 Introduction

Multiple Myeloma (MM) is a complex hematologic malignancy characterized by the uncontrolled proliferation of plasma cells within the bone marrow. Primarily affecting older adults, MM accounts for approximately 10% of all hematologic cancers. Despite advances in therapy, including proteasome inhibitors, immunomodulatory drugs, and monoclonal antibodies, MM remains largely incurable (1). Elderly patients, particularly those over the age of 70 or those with comorbidities, present a therapeutic challenge due to the higher likelihood of adverse events and the decreased ability to tolerate aggressive treatments like autologous stem cell transplantation (ASCT) (2).

MM represents a substantial and growing public health burden. According to GLOBOCAN estimates, approximately 176,000 new cases are diagnosed annually worldwide, with more than 60% occurring in individuals aged 65 years or older (3). As global populations continue to age, the absolute number of elderly MM patients eligible for non-transplant treatment pathways is projected to increase considerably, amplifying the pressure on healthcare systems to identify strategies that balance clinical effectiveness with economic sustainability (4). In the absence of a curative option and given the need for prolonged or continuous treatment, the cumulative economic burden associated with MM management is particularly pronounced in this population.

First-line therapies for elderly transplant-ineligible patients with MM typically include combination regimens such as bortezomib, melphalan, and prednisone (VMP) or lenalidomide and dexamethasone (Rd). The introduction of daratumumab, a monoclonal antibody targeting CD38, has expanded the options for treating this population, with regimens showing improved efficacy in clinical trials (5). However, the pharmacoeconomic implications of their widespread adoption as standard of care remain insufficiently characterized. Existing cost-effectiveness analyses have predominantly addressed pairwise comparisons of selected regimens, leaving a critical gap in the simultaneous evaluation of all major first-line options within a unified modeling framework (6, 7).

Cost-effectiveness analysis (CEA) provides a means of evaluating the value of new treatments by comparing their costs and clinical outcomes (8). For MM, such analyses are critical in guiding treatment decisions, particularly for healthcare systems facing budget constraints (9). This study aims to assess the cost-effectiveness of four first-line therapies for elderly MM patients or those ineligible for ASCT: bortezomib, melphalan, and prednisone (VMP); lenalidomide and dexamethasone (Rd); daratumumab combined with VMP (D-VMP); and daratumumab combined with Rd (DARA-Rd).

The Italian healthcare setting provides a particularly relevant context for this analysis. In Italy, approximately 5,000 new cases of MM are diagnosed each year, with a median age at diagnosis exceeding 70 years, rendering transplant-ineligible regimens the predominant therapeutic approach at the population level (10). The Italian National Health Service (INHS) operates under a universal coverage model in which reimbursement and pricing decisions for oncological agents are centrally governed by the Agenzia Italiana del Farmaco (AIFA) through a formal Health Technology Assessment (HTA) process (11). Within this framework, the incremental cost-effectiveness ratio constitutes a primary criterion for reimbursement eligibility, and the definition of an appropriate willingness-to-pay threshold remains a subject of active methodological and policy debate (12). Furthermore, drug acquisition costs are subject to regional negotiation and may vary substantially across hospital consortia, introducing additional uncertainty into economic evaluations and underscoring the need for analyses that explicitly account for price heterogeneity. These institutional and epidemiological characteristics collectively make Italy an informative and policy-relevant setting for the comparative cost-effectiveness assessment of first-line MM regimens, with findings that may carry implications for other healthcare systems sharing analogous structural features.

In this general context, pharmacoeconomic modelling offers a principled framework not only for comparing treatment costs and outcomes, but also for explicitly quantifying and propagating parameter uncertainty through probabilistic sensitivity analyses, thereby enabling robust and generalizable conclusions that extend beyond the specific assumptions of any single fixed-input scenario.

## 2 Materials and methods

This study employed a state-transition Markov model to evaluate the cost-effectiveness of four treatment regimens for elderly patients with multiple myeloma (MM) (13, 14). The simulated cohort included patients aged 70 years or older, as well as younger patients with comorbidities that preclude ASCT, who were newly diagnosed with MM according to the EHA–ESMO Clinical Practice Guidelines (15). Baseline patient characteristics are derived from pivotal clinical trials: the ALCYONE trial (706 patients) for VMP and D-VMP, and the MAIA trial (736 patients) for Rd and DARA-Rd (16–18). This approach ensures that the simulated cohort accurately reflects the demographic and clinical profile of elderly MM patients: 71.0 yrs. (40-93) in the treatment group of ALCYONE; 73.0 yrs. (50-90) in the treatment group of MAIA.

A line of therapy was defined as one or more cycles of a planned treatment program, which could consist of single-agent therapy, combination therapy, or a sequence of treatments administered in a structured manner. The first cycle of the first-line therapy was assumed to occur at time *t* = 0, with all newly diagnosed patients considered immediately eligible to initiate first-line treatment.

Each cycle was defined as 42 days for VMP-based therapies. For Rd-based regimens, which have 28-day cycles according to trial protocols, the cycle length was standardized to 42 days for the purposes of cost and utility calculations. The total simulation horizon, defined as the overall duration of the simulation, comprises 45 cycles (approximately 5.25 years), a value consistent with both the median overall survival of MM reported in the literature and the survival estimates from the ALCYONE and MAIA trials. We acknowledge that a lifetime horizon is frequently adopted in oncology economic evaluations. However, we deliberately selected a trial-informed finite horizon to minimize uncertainty associated with long-term extrapolation of survival, costs, utilities, and treatment effects beyond the available evidence base. Regarding the cycle-length standardization, this is a deterministic accounting convention: per-cycle costs and utilities for the 28-day regimens are rescaled proportionally to the 42-day cycle, which conserves both quantities per unit of calendar time and does not introduce any estimable parameter. It is therefore held fixed rather than treated as a source of structural uncertainty in the probabilistic sensitivity analysis, in which the per-cycle cost and utility inputs are instead sampled from their prior distributions (Subsection 2.8).

The model evaluated pre-specified treatment sequences rather than isolated first-line regimens alone. For each first-line strategy, a fixed second-line therapy was assigned after relapse/progression in order to capture the downstream impact of post-relapse treatment on costs and health outcomes. The second-line therapies considered in the simulation were specified as follows: VMP → ISA-Kd, Rd → ISA-Pd, D-VMP → KRd, and DARA-Rd → VPd.

These sequences were defined *a priori* according to clinical plausibility, prior treatment exposure, and consistency with current evidence-based recommendations for the management of relapsed/refractory multiple myeloma (15, 19). In accordance with EHA–ESMO Clinical Practice Guidelines, the selection of salvage therapy should consider the mechanism of action of prior treatments, the duration of response to first-line therapy, and the avoidance of cross-resistance (15). Applying these principles, the following rationale was adopted for each sequence. VMP-treated patients were assigned ISA-Kd at relapse, as they had not been previously exposed to an anti-CD38 monoclonal antibody, rendering isatuximab-based therapy a clinically appropriate and guideline-consistent salvage option. Patients treated with D-VMP were assigned KRd, reflecting prior exposure to both daratumumab and bortezomib; in this context, a carfilzomib- and lenalidomide-based regimen represents a recommended option that avoids reintroduction of agents to which resistance may have developed. Patients treated with Rd were assigned ISA-Pd, consistent with guideline recommendations favouring pomalidomide-based regimens as post-relapse therapy following lenalidomide-containing first-line treatment, given the well-established cross-resistance between lenalidomide and pomalidomide at the level of cereblon binding (19, 20). Finally, patients treated with DARA-Rd were assigned VPd, reflecting prior exposure to both daratumumab and lenalidomide; in this setting, a bortezomib- and pomalidomide-based regimen without an anti-CD38 component represents a clinically plausible salvage strategy consistent with current practice recommendations.

Therefore, the resulting costs, QALYs, and ICERs should be interpreted as referring to complete treatment pathways conditional on the specified post-relapse assumptions, rather than as estimates of the cost-effectiveness of first-line drug regimens in isolation. Although additional lines of therapy are considered in clinical practice, the analysis is restricted to second-line treatment, in accordance with the time horizon adopted in the simulation. Further details on the scheduling of therapies are provided in the **Supplementary Material**.

This cost-effectiveness analysis was conducted and is reported in accordance with the Consolidated Health Economic Evaluation Reporting Standards 2022 (CHEERS 2022) checklist (21). CHEERS 2022 was adopted as a reporting framework rather than as a methodological guideline. A completed checklist is provided in the **Supplementary Material**.

### 2.1 Model states and graphical representation of the state-transition structure

The state-transition Markov model simulates patient progression across six mutually exclusive health states: active therapy [T], infection [I], hematologic complications [H], maintenance [M], relapse [R], and death [D]. The structure of the Markov model is represented as a collection of oriented state-transition diagrams, in which nodes correspond to mutually exclusive health states and directed edges represent admissible one-step transitions between states across consecutive cycles. This representation constitutes the standard graphical formalism for discrete-time cohort state-transition models in health economic evaluation (13, 14, 22).

**Figure 1:**
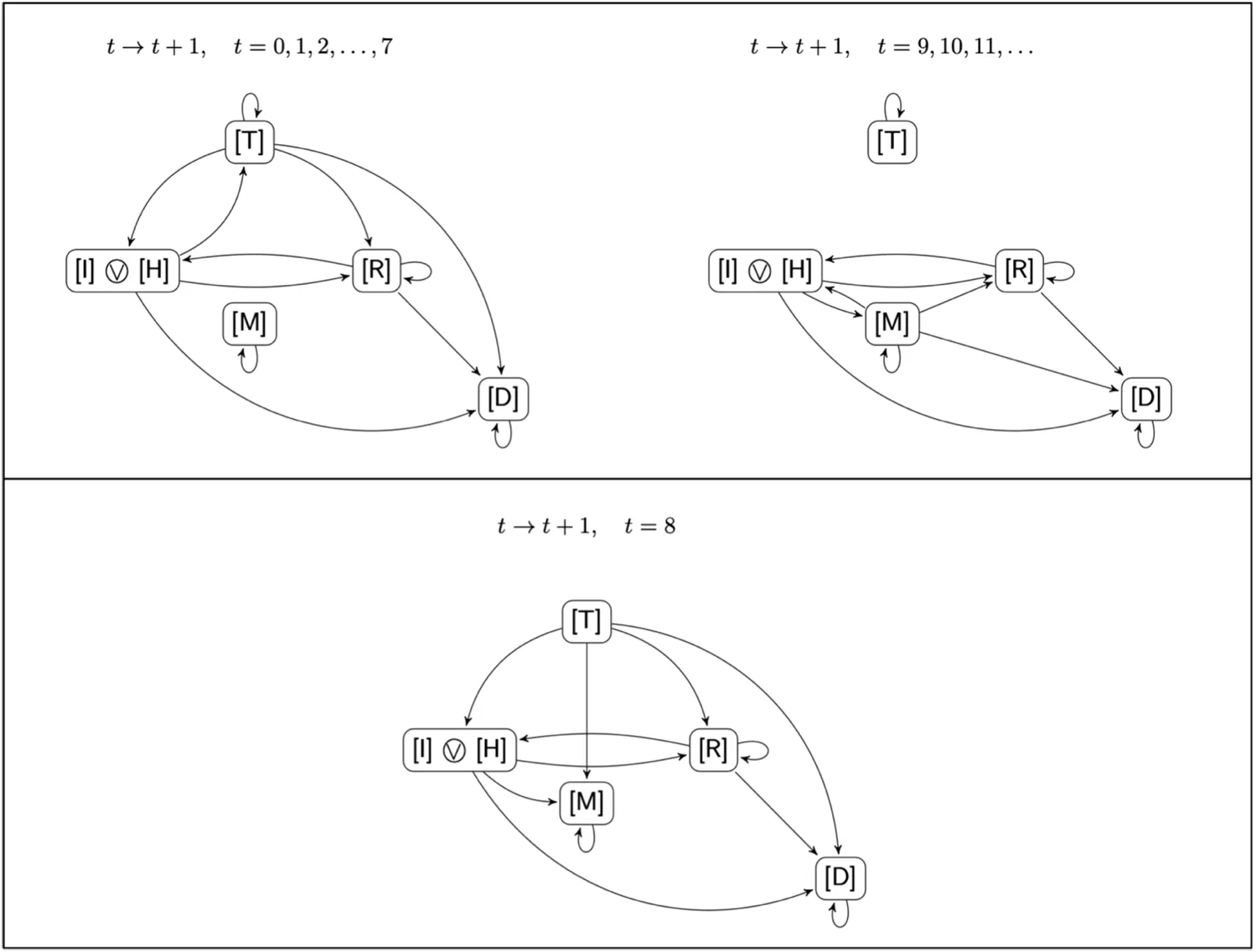
State-transition diagrams of the Markov model, shown for VMP/D-VMP as a representative example. Nodes denote the mutually exclusive health states of the model — active first-line therapy [T], infection [I], hematologic complications [H], maintenance [M], relapse/progression [R], and death [D] — and directed edges denote the admissible one-step transitions between consecutive cycles; a self-loop indicates that patients may remain in the same state over the following cycle. The three panels correspond to three distinct phases of the simulation, which differ in their admissible transitions. (i) Upper-left panel — active first-line therapy (*t* = 0–7): patients may remain in [T], experience a transient adverse-event state ([I] or [H]), progress to relapse [R], or die [D]. The maintenance state [M] is not yet reachable and is shown in isolation with a unit self-loop. (ii) Lower panel — final cycle of first-line therapy (*t* = 8): at the beginning of this cycle the active-treatment state [T] loses its self-loop and all patients still in [T] are redistributed across the remaining reachable states, so that [T] becomes isolated from t = 9 onward. (iii) Upper-right panel — maintenance phase (*t* ≥ 9): patients who have neither progressed nor died transition to the maintenance state [M], from which relapse [R] and death [D] remain admissible. States [I] and [H] are drawn within a single node for visual compactness but represent two separate, mutually exclusive health states: within any given cycle a patient may occupy [I] or [H], but never both simultaneously. They share an identical set of outgoing transitions, which is what allows them to be depicted jointly without loss of information. Differences applicable to Rd/DARA-Rd are described in the main text.

The diagrams are organized according to three distinct phases of the simulation. For illustrative purposes, the following description refers to the state-transition structure of the model for VMP/D-VMP. The upper-left panel of **Figure 1** describes the admissible transitions during the first eight cycles of first-line therapy (*t* = 0, 1, …, 7), in which patients may remain in the active treatment state [T], experience a transient adverse-event state ([I] or [H]), progress to relapse [R], or die [D]. During this phase, the maintenance state [M] is isolated, with a self-loop corresponding to a transition probability of one, as no patient is expected to enter [M] prior to the completion of first-line therapy. The upper-right panel describes the transition structure following the completion of first-line therapy (*t* = 9, 10, 11, …) during which patients who have neither died nor progressed enter the maintenance phase. The lower panel of **Figure 1** describes the transitional cycle *t* = 8, corresponding to the administration of the final cycle of first-line therapy, at the beginning of which the state [T] loses its self-loop and all patients are redistributed to the remaining reachable states, with [T] becoming isolated at *t* = 9.

With respect to states [I] and [H], it should be noted that, although they are represented within a single node in the diagrams for clarity of exposition, they constitute two distinct and mutually exclusive health states: a patient may occupy state [I] XOR state [H] within any given cycle, but not both simultaneously. The permitted transitions out of [I] and [H] are identical, which justifies their joint graphical representation without loss of generality.

### 2.2 Transition probabilities

In the most general setting, transitions between states are non-stationary and are described by a transition probability matrix *P_t_* that varies with simulation time *t*. This defines a Markov chain over the state space that evolves accordingly. The update equations governing the probability distribution over states follow the standard recursion rules of Markov chain theory (14). In what follows, we briefly describe the rationale underlying the computation of these transition probabilities.

#### 2.2.1 Infections [I] and hematologic disorders [H]

In any cycle, due to multifactorial immunodeficiency caused by the disease itself and the treatment regimens, a grade 3–4 infection may occur, modeled with a transient state in which the patient remains for the duration of a single cycle. Infection incurs significant additional costs associated with antibiotic therapy and hospitalization (in addition to the cost of antineoplastic therapy). In principle, infections can lead to discontinuation of therapy. However, this adverse event occurs with a very low probability.

The states [I] and [H] should be interpreted as transient one-cycle adverse-event states rather than as persistent disease states. They were introduced to explicitly capture severe complications requiring hospital-based management and associated with distinct resource-use profiles. This formulation allowed infection-related and hematologic-complication-related costs, together with the corresponding disutilities, to be assigned only to patients experiencing the relevant adverse event.

For VMP/D-VMP, the transition probabilities of developing an infection at the beginning of each subsequent 42-day cycle were estimated based on the data reported for infectious complications in (23), assuming a reference period corresponding to the median follow-up of 40.1 months and computing an average rate of the most frequent grade 3–4 respiratory infections. For Rd/DARA-Rd, an analogous computational framework was adopted, using data reported in (24), referring patients with a median follow-up of 56.2 months.

Similarly, neutropenia, thrombocytopenia, and anemia represent common hematologic complications associated with anti-neoplastic therapies. It is important to note that a substantial overlap exists between patients experiencing these conditions and those developing infections. For instance, patients with severe neutropenia are at markedly increased risk of bacterial infections. Nevertheless, these two categories of adverse events—namely, infections and hematologic dysfunctions—entail distinct cost structures, reflecting the different therapeutic interventions required. In this setting, it is assumed that the [H] state represents a transient condition that can be resolved within a single cycle through appropriate treatment, and that the occurrence of such adverse events does not lead to discontinuation of anti-MM therapy. The data sources used to estimate the probabilities of developing hematologic complications at the beginning of each subsequent cycle are the same as those employed for infectious complications.

#### 2.2.2 Disease relapse [R] and death [D]

Cycle-specific transition probabilities toward states [R] and [D] were derived from the estimated Kaplan-Meier curves published in the ALCYONE and MAIA trials, together with the number of patients at risk at evenly spaced time points (23, 24). The dataset containing the coordinates of the survival functions for each treatment arm was extracted using the digitizing software WebPlotDigitizer^1^.

Appropriate inversion algorithms were employed to reconstruct overall survival (OS) and progression-free survival (PFS) from the digitized Kaplan-Meier curves. Cycle-specific transition probabilities were then derived by fitting parametric survival models to the reconstructed data. For each endpoint and treatment strategy, exponential and Weibull specifications were compared using the Bayesian Information Criterion (BIC), and the model with the lowest BIC was selected. The probability of transitioning to the death state [D] was estimated from OS data, whereas the probability of transitioning to the relapse/progression state [R] was derived from PFS data.

The transition probability to the state [M] (maintenance), whenever this state is reachable, has been computed residually with respect to the remaining transition probabilities, by taking the complement to 1 of their sum. Additionally, the Markov cohort model employs a Markov chain to simulate cohort evolution across multiple states, each of which can be reached at most once within a given cycle. This feature constrains the specification of transition probabilities. For instance, the probability that a patient receiving treatment at the start of a given cycle will relapse at the beginning of the subsequent cycle has been consistently computed under the assumption that the patient survives until the start of the next cycle and therefore is not among those who die during the current cycle.

### 2.3 Simulation setting

The progression of the cohort across health states was simulated using the non-stationary Markov cohort model described above. The scheduling and dosing of each treatment regimen were specified as follows: (a) VMP: nine 42-day cycles with predefined dosages of bortezomib, melphalan, and prednisone; (b) D-VMP: nine 42-day cycles combining daratumumab with VMP, followed by a maintenance phase initiated after cycle 9; (c) Rd: continuous administration of lenalidomide and dexamethasone; (d) DARA-Rd: 28-day cycles (mapped to 42-day intervals within the model) with a predefined dosing schedule.

All analyses were implemented entirely in the R statistical environment (version 4.5.2) within RStudio, using a tidyverse-based workflow; no spreadsheet software was used at any stage, including for cost-data organization or table generation. Reconstruction of the published Kaplan–Meier curves and the subsequent parametric survival modeling relied on the reconstructKM, survival, survminer, and flexsurv packages. The Markov cohort simulation, the fixed-input base-case analysis, and the probabilistic sensitivity analysis were implemented using darthtools and dampack. Data manipulation and reshaping were performed with tidyverse and reshape2, and tabular outputs were generated programmatically with xtable; figures were produced with ggplot2 and the ggpubr, ggsci, and ggthemes extensions. To ensure full reproducibility, the complete source code has been deposited on a dedicated Zenodo host repository^2^. The full analytical pipeline, from data input through simulation to output generation, is therefore scripted and reproducible from the source code.

### 2.4 Utilities

Within the model, utilities encode preferences over health conditions, with more desirable states assigned higher values. These quantities are defined on a cardinal scale ranging from 0 to 1, where 0 corresponds to death and 1 denotes perfect health (25, 26). In practice, utility values are translated into QALYs (Quality-Adjusted Life Years) by weighting them according to the fraction of a year spent in each health state (e.g., 1 QALY corresponds to one year lived in perfect health). QALYs provide a composite measure that integrates survival duration and health-related quality of life (QoL), enabling coherent comparisons of cost-effectiveness across alternative therapeutic strategies (27).

The summary metric used for comparing two competing interventions is the incremental cost-effectiveness ratio (ICER), defined as the ratio between the difference in expected total costs and the difference in expected health outcomes, measured in QALYs, as given below:


$$ICER_{A\to B}=\frac{C_{B}-C_{A}}{U_{B}-U_{A}},$$

where *C_B_* and *C_A_* represent the average total costs associated with the two strategies, and *U_B_* and *U_A_* denote the corresponding average total utilities expressed in QALYs (28).

For VMP/D-VMP, utility values for the states [T], [M], and [R] are derived from published evidence based on the EQ-5D-5L instrument, relying exclusively on quality-of-life data collected from the same cohort of patients enrolled in the ALCYONE trial (29). Analogously, utility estimates for Rd/DARA-Rd are obtained from the same measurement scale using data from patients included in the MAIA trial (30). For transitions to the states [I] and [H], additional disutilities associated with the physical and psychological burden of acute adverse events are incorporated.

Values for the additional transition-related disutilities were sourced from the existing literature. In particular, those associated with states [I] and [R] were derived from (31), and represent incremental disutilities relative to baseline corresponding to at least one infection and to disease progression, respectively, in a cohort of patients treated with Selinexor for relapsed/refractory diffuse large B-cell lymphoma. Notably, more than 60% of patients in this cohort were aged ≥65, and the reported values were obtained using the EQ-5D-5L instrument.

The disutility associated with state [H] was obtained from (32), reflecting the additional burden of febrile neutropenia in patients with non-small cell lung cancer undergoing chemotherapy. These estimates were based on the EORTC QLQ-C30 scale (33).

The disease-state utilities for [T], [M], and [R] were obtained directly from the multiple myeloma populations of the ALCYONE and MAIA trials, whereas only the incremental disutilities associated with acute infectious and hematologic adverse events were transferred from other oncologic and hematologic cohorts. To the authors’ knowledge, no published study reports preference-based disutility estimates for grade 3–4 infectious or hematologic complications specifically in multiple myeloma. The selected sources were therefore chosen to maximize instrument and demographic comparability, favouring the same EQ-5D-5L instrument and an elderly-enriched population for the infection- and progression-related disutilities. These parameters were sampled from their prior distributions within the probabilistic sensitivity analysis, so that the uncertainty arising from cross-population transfer is explicitly propagated to the results.

### 2.5 Costs and perspective

Costs were evaluated from the perspective of the Italian public payer, including medical expenditures associated with drug and medical supply consumption, laboratory services, medical equipment, and professional fees (34). These costs are sustained by the Italian Regions and ultimately by the Italian Government through the Italian National Health Service (INHS). Direct and indirect out-of-pocket expenses incurred by patients were not considered, as they are generally negligible relative to the overall medical costs covered by the INHS. Adverse event-related costs, such as those incurred from managing infections or hematologic toxicities, were considered, along with the costs of second-line therapies following relapse. Further details on unit costs, treatment-cycle cost calculations, and the main structural parameters used in the fixed-input base-case analysis are provided in Section 2 of the **Supplementary Material**.

A table reporting the complete set of unit costs employed in this study is provided in the companion paper (35). The final price per single pack reported in that reference corresponds to the price negotiated in 2023 by the Hospital Consorziale Policlinico in Bari, Apulia, Italy, through direct agreements with manufacturers. These prices may vary across hospitals and Regions; for this reason, they are treated as input quantities subject to uncertainty within the probabilistic simulation described in Subsection 2.8.

For the computation of ICERs, all costs were expressed in euros for the selected reference year (2023). Future costs and health outcomes were discounted to account for time preference. In the fixed-input base-case analysis, in accordance with Italian pharmacoeconomic recommendations, both future costs and QALYs were discounted at an annual rate of 3% (11). In the absence of further information supporting differential discounting, the same discount rate was applied to both costs and health outcomes. Alternative discount-rate assumptions were explored in probabilistic sensitivity analyses to assess the robustness of the results.

### 2.6 Outcomes

The primary outcome measure was cost-effectiveness, expressed as the incremental cost-effectiveness ratio (ICER). Secondary endpoints included PFS, OS, and the incidence of treatment-related adverse events. As previously noted, QALYs were computed by assigning utility weights to each health state and multiplying them by the duration of each cycle, expressed as a fraction of a year.

### 2.7 Internal and external consistency

Internal validation was performed by checking the consistency between the conceptual Markov structure and its computational implementation, including transition probabilities, state occupancy, cycle-specific costs, utilities, and accumulated QALYs. Additional checks were conducted to ensure that simulated cohort proportions remained within admissible bounds and that model outputs were numerically stable across probabilistic simulations. Probabilistic sensitivity analysis was used to assess parameter uncertainty and should therefore be interpreted as an uncertainty analysis rather than as a formal validation exercise.

The face and content validity of the model was further corroborated by the direct involvement of the clinical co-authors, senior specialists in the management of multiple myeloma, whose expertise informed the structural development of the model throughout. Their appraisal encompassed the conceptual architecture of the model, including the specification of the health states, the admissible inter-state transitions, and the definition of the first- and second-line treatment sequences, thereby ensuring its clinical plausibility and its consistency with prevailing therapeutic practice.

External validation was not performed because no independent dataset was available that reproduced the full set of treatment strategies, model states, and follow-up conditions considered in the analysis.

### 2.8 Probabilistic sensitivity analysis

If based on fixed input values, the results themselves may be subject to criticism due to their limited generalizability, as such input parameters may vary across settings or certain cost components may be absent depending on the structure of the healthcare system. A more robust approach to assessing generalizability consists in explicitly accounting for uncertainty in both parameter estimates and cost/utility values. Probabilistic sensitivity analysis (PSA) addresses this issue by repeatedly sampling model parameters and inputs from their respective prior probability distributions, rather than relying exclusively on point estimates such as means or medians (36).

For example, cost components were modeled using a Γ(*α*, *s*) distribution, with the parameters *α* (shape) and *a* (scale) estimated via the method of moments (22). The discount rates for both costs and utilities were specified through a discrete Uniform distribution (Categorical), with values ranging from 1.5% to 7.5% in increments of 0.1%.

For a fixed negotiated drug price per pack *μ*, a Gaussian prior was adopted with mean *μ* and standard deviation σ, calibrated such that *μ* ± 3σ corresponds to a variation of approximately ±20% around *μ*. This implies σ=(0.2/3)μ, ensuring that most simulated prices lie within this range. Similarly, transition probabilities and utility parameters were sampled from suitable distributions.

Cost-effectiveness acceptability curves (CEACs) are used to summarize the output of the probabilistic sensitivity analysis (PSA) (37). For their construction, the net monetary benefit (NMB) can be considered:


$$NMB_{i\left(j\right)}\left(\lambda │\theta_{i\left(j\right)}\right)=\lambda U_{i}\left(\theta_{i\left(j\right)}\right)-C_{i}\left(\theta_{i\left(j\right)}\right),\;i=1,2,3,4,$$

where λ denotes the WTP threshold expressed in Euro, and $NMB_{i\left(j\right)}\left(\lambda │\theta_{i\left(j\right)}\right)=\lambda U_{i}\left(\theta_{i\left(j\right)}\right)-C_{i}\left(\theta_{i\left(j\right)}\right),\;\;\;\;i=1,2,3,4,$ and $C_{i}\left(\theta_{i\left(j\right)}\right)$ represent, respectively, the average total QALYs and average total costs associated with therapeutic strategy *i* for a given simulated parameter set $\theta_{i\left(j\right)}$. To estimate the cost-effectiveness probability, for each simulation and for each value of λ, we first identify the strategy that yields the highest net monetary benefit within the simulated scenario *j*:


$$\text{best}\;NMB_{\left(j\right)}\left(\lambda │\theta_{i\left(j\right)}\right)={\text{argmax}}_{i=1,2,3,4}NMB_{i\left(j\right)}\left(\lambda │\theta_{i\left(j\right)}\right),\;$$

and the value of the curve $CEAC_{i}\left(\lambda \right)$ is then estimated as the proportion of simulations in which strategy *i* is optimal, that is, attains the highest NMB for a given value of λ.

## 3 Results

### 3.1 Fixed-input base-case analysis

According to the BIC values, the exponential model was selected for OS in all four treatment strategies, resulting in transition probabilities toward [D] that are constant over cycles. Conversely, the selected PFS models implied increasing relapse probabilities for VMP/D-VMP and constant relapse probabilities for Rd/DARA-Rd. The constant OS transition probabilities should therefore be interpreted as the result of the selected parametric fit within the finite trial-informed time horizon, rather than as an assumption of lifetime constant mortality hazards.

**Figure 2:**
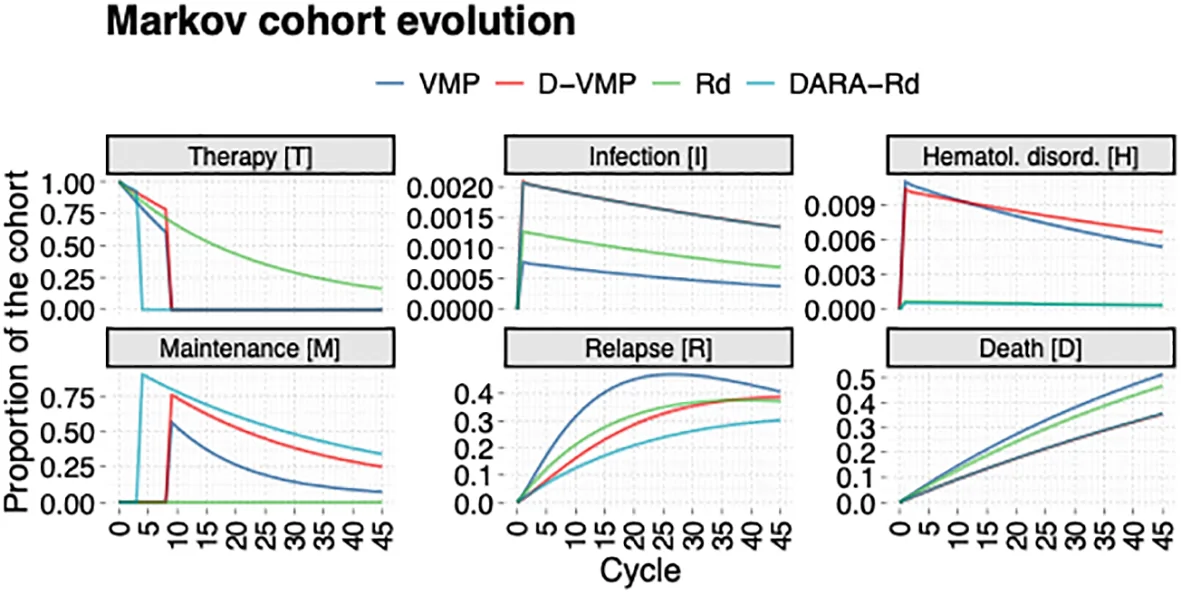
Temporal evolution of the simulated patient cohort based on the estimated transition probabilities. At *t* = 0, all patients occupy the state [T] and receive the first therapy cycle. In cases where the red line corresponding to D-VMP is not visible, because it overlaps with the corresponding trajectory of VMP.

Given the parameter values specified for the base-case analysis, **Figure 2** shows the temporal evolution of the patient cohort, based on the transition probabilities governing movements across health states.

**Figure 3:**
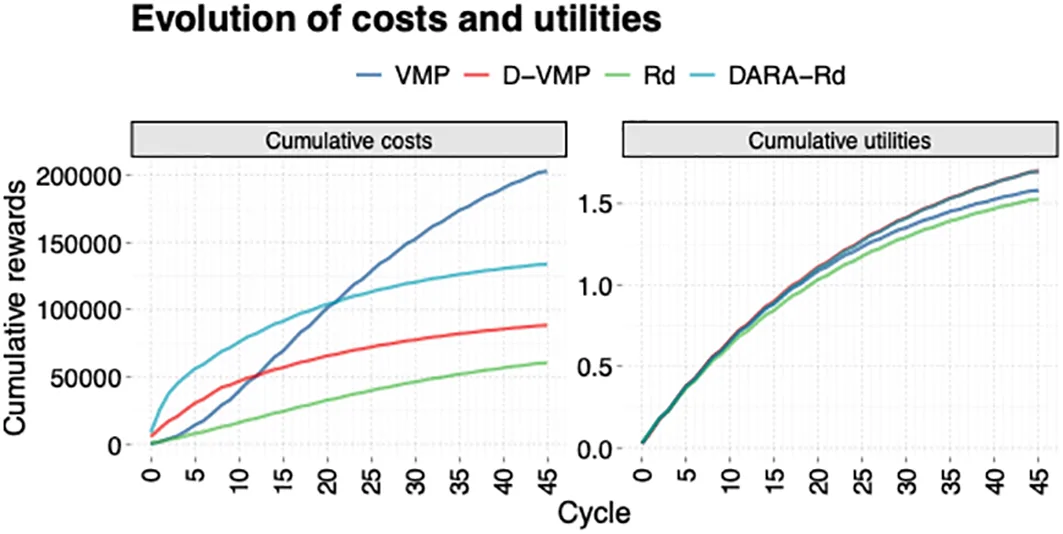
Temporal evolution of cycle-specific average costs and utilities. For DARA-Rd and D-VMP, the cumulative utility curves are nearly superimposed. Both costs and utilities were discounted at an annual rate of 0.030 (3%).

**Figure 3** reports the temporal trajectories of utilities and costs associated with each treatment strategy, showing how these quantities evolve over treatment cycles and subsequent follow-up. For each regimen, cost and utility profiles can be compared dynamically over time.

VMP/D-VMP exhibit a progressive increase in costs, primarily driven by cumulative drug administration and the management of treatment-related complications. In contrast, utility remains relatively stable, reflecting a broadly consistent quality of life throughout the treatment cycles. In particular, D-VMP is characterized by a substantial initial cost burden, with costs rising during the early cycles and subsequently stabilizing as treatment advances. Utility shows a modest decline over time.

Rd exhibits a more stable cost trajectory compared to VMP/D-VMP, with utility remaining relatively constant over time. In contrast, DARA-Rd entails higher initial costs due to the inclusion of daratumumab, with expenditures continuing to increase over time because of sustained treatment and associated management.

The cost-utility analysis is summarized in **Table 1**, illustrating the fixed-parameter scenario within an extended-dominance framework (38). Overall, Rd and D-VMP emerge as non-dominated (ND) strategies, as they provide greater benefits relative to the available alternatives. Although D-VMP is more costly than Rd, it also yields a higher number of QALYs. The incremental cost-effectiveness ratio (ICER) of D-VMP relative to Rd is approximately €160,000, implying a substantially high willingness-to-pay threshold for cost-effectiveness.

**Table 1:** Cost-utility analysis of four first-line multiple myeloma regimens: incremental cost-effectiveness and dominance status for the fixed-parameter scenario.

Strategy	Costs (€)	QALYs	Incremental costs (€)	Incremental QALYs	ICER (€/QALY)	Status
Rd	60,483	1.53	–	–	–	ND
D-VMP	88,331	1.70	27,848	0.17	160,804	ND
DARA-Rd	133,770	1.70	–	–	–	D
VMP	203,006	1.58	–	–	–	D

By contrast, DARA-Rd and VMP are dominated (D) strategies, as they incur higher costs while delivering lower utility. It is noteworthy that the VMP arm, characterized by the lowest PFS, progressively reflects the increasing burden of patients experiencing disease relapse and subsequently requiring second-line isatuximab-Kd immunotherapy. This regimen is administered beyond 9 cycles and entails costs comparable to daratumumab-based therapy, owing to the combined expenses of isatuximab and carfilzomib.

QALYs were not determined exclusively by time spent in progression-free survival, but also incorporated treatment-specific adverse-event burden, including severe infections and hematologic complications. Therefore, within the selected finite time horizon, the clinical benefit associated with improved disease control may be partially offset by disutilities related to treatment intensity and acute toxicity.

### 3.2 Results from the PSA

**Figure 4:**
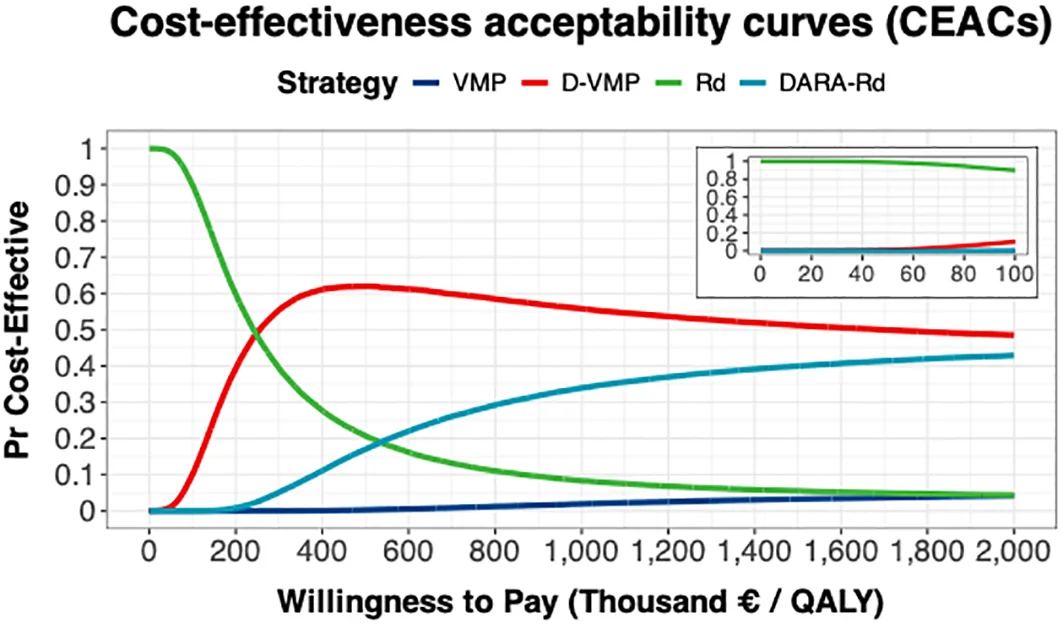
Cost-effectiveness acceptability curves (CEACs) showing the probability that each strategy is cost-effective across a range of willingness-to-pay (WTP) thresholds. The main panel reports the CEACs over the full WTP range from €0 to €2,000,000 per quality-adjusted life year (QALY) gained, evaluated using increments of €500, whereas the inset in the upper-right corner provides a magnified view of the policy-relevant range between €0 and €100,000/QALY. The probability of cost-effectiveness for each therapeutic strategy is estimated as the proportion of probabilistic simulations in which that strategy is optimal, that is, the strategy attaining the highest net monetary benefit (NMB), for a given WTP threshold.

To perform the PSA on model inputs, *N* = 50,000 parameter sets were randomly sampled, and the corresponding total discounted costs and QALYs of the competing strategies were computed for each realization. **Figure 4** contains the CEAC curves, representing the probability that a given intervention is cost-effective across a range of willingness-to-pay (WTP) thresholds, that is, the maximum monetary value that society or a patient is willing to pay for one additional unit of QALY.

Increasing the intrinsic variability of the model parameters shifts upward the threshold beyond which D-VMP becomes the most likely cost-effective strategy. In particular, the CEACs indicate that Rd is more likely to be cost-effective for willingness-to-pay thresholds below €250,000 per additional QALY. Above this level, D-VMP emerges as the strategy offering the most favorable trade-off between economic cost and patient benefit.

The inset reported in **Figure 4** provides a clearer visualization of the CEACs within the most policy-relevant WTP range. In this magnified region, D-VMP shows a progressively increasing probability of being cost-effective and becomes more favorable than both VMP and DARA-Rd already at a WTP threshold of approximately €50,000/QALY. This pattern indicates that, even within relatively conservative willingness-to-pay values, D-VMP tends to dominate these two alternatives in probabilistic terms. However, the comparison with Rd remains more nuanced and depends on the specific WTP threshold considered, as reflected by the relative position of the corresponding CEACs.

## 4 Discussion and conclusions

This study evaluated the cost-effectiveness of four first-line regimens for transplant-ineligible or elderly patients with newly diagnosed MM. Within the time horizon and structural assumptions of the Markov model, Rd and D-VMP emerged as the only non-dominated strategies, whereas DARA-Rd and VMP were dominated. The probabilistic analysis supports a more nuanced interpretation: Rd appears more likely to be cost-effective at lower willingness-to-pay thresholds, while D-VMP becomes competitive only when the threshold exceeds approximately €160,000 per QALY from the perspective of the Italian NHS. In this sense, D-VMP cannot be regarded as uniformly cost-effective; rather, its economic attractiveness depends on relatively high WTP thresholds and on the assumptions governing survival, relapse, and post-progression management. Accounting for parameter uncertainty through probabilistic simulation, which increases the intrinsic variability of the model inputs, shifts this threshold upward to approximately €250,000.

It should be noted that Italy has not adopted an explicit cost-effectiveness threshold, and AIFA does not rely on a fixed willingness-to-pay value when negotiating price and reimbursement; empirically, the incremental cost-effectiveness ratios of medicines reimbursed by the INHS cluster around €30,000/QALY, approximately equal to national GDP per capita (39). Italian methodological guidance has conventionally referred to an acceptability range of approximately €25,000–€40,000/QALY (40). Against these benchmarks, the ICER of €160,804/QALY estimated for D-VMP, and the threshold of approximately €250,000/QALY emerging from the probabilistic analysis, clearly exceed the values typically regarded as cost-effective in the general medical context.

This positioning must, however, be interpreted in light of the oncological and end-of-life nature of the decision problem. Several jurisdictions explicitly apply higher willingness-to-pay thresholds to life-extending cancer therapies; NICE, for instance, evaluates end-of-life and rare-disease treatments against a threshold of approximately £50,000/QALY, well above its conventional £20,000–£30,000/QALY range (41). More importantly, the value we obtained is consistent with—and indeed more favorable than—most published cost-effectiveness estimates for daratumumab-based frontline therapy in transplant-ineligible myeloma. Using MAIA-derived data, first-line daratumumab has been associated with an ICER as high as US $618,018/QALY relative to reserving the agent for later lines, whereas ALCYONE-based analyses have reported an ICER of approximately US $226,836/QALY for D-VMP versus VMP (6, 42). Analyses comparing daratumumab-based triplets with bortezomib-based regimens have reported lower ICERs, in the range of US $82,000–$90,000/QALY, depending on the comparator and setting (43). Our estimate of €160,804/QALY therefore falls toward the lower-to-middle portion of the range documented internationally for this drug class and population, reinforcing the conclusion that the economic profile of daratumumab-based frontline therapy appears largely driven by acquisition costs.

Our results align with the broader health-economic literature on MM. Previous reviews have consistently shown that newer agents tend to improve both survival and quality-adjusted survival, but at the cost of substantial incremental expenditures, often leading to ICERs above commonly accepted thresholds (7). This general pattern is reflected in our findings, where clinically superior daratumumab-based regimens do not automatically translate into favorable economic value.

The comparison between Rd and VMP is also consistent with earlier evidence. Usmani et al. reported that Rd may represent a cost-effective alternative to VMP in transplant-ineligible patients, with gains in both life-years and QALYs at an ICER considered acceptable in the U.S. setting (44). A similar pattern emerges here: Rd occupies the most favorable position at lower WTP thresholds and remains on the efficiency frontier, while VMP is consistently dominated over the modeled horizon.

The role of daratumumab-based frontline therapies remains more debated, and our findings should be interpreted within this broader context. Li et al. suggested that D-VMP could be cost-effective versus VMP at a WTP threshold of $150,000/QALY in the U.S. setting (45). In contrast, Patel et al. showed that first-line daratumumab may not be cost-effective under current pricing, highlighting the need for substantial price reductions (6). Similar conclusions were reached in other settings: Wong et al. found that DARA-Rd was not cost-effective in Singapore, while Narsipur et al. reported a very low probability that DARA-Rd or VRd (bortezomib + lenalidomide + dexamethasone) would be cost-effective compared with Rd (46, 47). More recently, Bayani et al. emphasized again that the economic sustainability of first-line daratumumab critically depends on pricing strategies (43).

Taken together, these studies indicate that the economic value of daratumumab-based combinations is highly context-dependent, being strongly influenced by drug acquisition costs, modeling horizon, survival extrapolation, and assumptions on subsequent treatments. This consideration is directly relevant here. In our model, DARA-Rd achieved the most favorable clinical outcomes in terms of disease control yet remained economically unattractive because the additional costs were not offset by sufficient incremental QALYs. Beyond survival outcomes, differences in utility trajectories deserve consideration. The relatively stable utility profile observed for Rd may be related to the continuous administration schedule and lower treatment burden, whereas the progressive improvement observed under D-VMP may suggest that the benefits associated with prolonged disease control outweigh the impact of treatment-related toxicity over the selected time horizon (29). Similarly, the apparent discrepancy between the superior PFS profile of DARA-Rd and its slightly lower QALY estimate compared with D-VMP underscores the importance of incorporating adverse-event burden and treatment-related disutilities into cost-effectiveness assessments (30). This does not call into question its clinical benefit; rather, it suggests that the magnitude of that benefit may not justify the associated costs under current conditions.

Another important aspect concerns treatment sequencing. Economic evaluations that consider full treatment pathways, rather than isolated regimens, have shown that innovative sequences can yield meaningful survival gains, but often at costs exceeding standard thresholds unless pricing adjustments are introduced (48). This is particularly relevant in our analysis, where the cost profile of each first-line strategy is also shaped by the second-line therapy assigned after relapse. Consequently, the results should be interpreted as reflecting the combined effect of first-line efficacy, relapse dynamics, and downstream treatment costs.

Several limitations should be acknowledged. Transition probabilities for relapse and death were reconstructed from digitized Kaplan–Meier curves and extrapolated parametrically, a standard but potentially sensitive procedure with respect to long-term survival assumptions. A partitioned survival framework, in which state membership is derived directly from the progression-free and overall survival curves, represents a recognized alternative to the state-transition structure adopted here (49). We opted for an explicit transition model because the analysis required transient adverse-event states—severe infections and hematologic complications—with their own costs and disutilities, which fall outside the progression-based partition underlying a partitioned survival model and are more naturally represented, and kept mutually coherent with the survival dimensions, within a transition structure. Moreover, since the pivotal trials report only graphical Kaplan–Meier estimates, the survival curves required digitization and reconstruction irrespective of the framework chosen; a partitioned survival model based on the same evidence would therefore not have eliminated this source of uncertainty.

Post-relapse treatment was simplified by assigning a single second-line strategy to each first-line regimen, whereas real-world pathways are more heterogeneous. In addition, patient frailty was not explicitly modeled, despite its well-known impact on treatment tolerability, outcomes, and healthcare utilization in this population. Future research should extend the present framework by incorporating frailty stratification, toxicity profiles, and real-world effectiveness data.

Also, some concerns regarding the parametric modelling of OS should be highlighted. Although the exponential specification was selected by BIC for all treatment strategies, constant mortality hazards may be restrictive in elderly patients over very long-time horizons. This limitation is partly mitigated using a finite time horizon anchored to the available trial follow-up, rather than by adopting a lifetime extrapolation requiring stronger long-term assumptions.

Another limitation of the analysis is that the four first-line regimens were informed by two separate pivotal trials rather than by a single head-to-head study. Since the four regimens were not compared within a single randomized trial and the evidence network was not fully connected, no formal indirect treatment comparison was performed. Therefore, the analysis should be interpreted as a model-based cost-effectiveness comparison conditional on the trial-derived inputs, rather than as a direct head-to-head comparative effectiveness analysis. Moreover, although ALCYONE and MAIA refer to broadly comparable populations of transplant-ineligible patients with newly diagnosed multiple myeloma, differences in trial design, control arms, follow-up, and patient characteristics may have contributed to between-trial heterogeneity.

Although the present analysis was designed to remain closely anchored to the available follow-up duration of the pivotal ALCYONE and MAIA trials in order to limit uncertainty related to long-term extrapolation, we acknowledge that the selected finite time horizon, the absence of formal indirect treatment adjustment, and the use of parametric survival assumptions may influence the estimated ICERs and QALY differences across treatment strategies. Therefore, the results should be interpreted as model-based estimates conditional on the adopted structural and clinical assumptions, rather than as definitive comparative-effectiveness conclusions.

Lastly, the economic evaluation was based on fixed second-line assignments after relapse. Therefore, the model estimates the cost-effectiveness of pre-specified treatment sequences rather than the isolated effect of first-line regimens alone. Since second-line regimens differ substantially in acquisition and administration costs, these assumptions may influence total costs and ICERs. In particular, the total cost associated with the VMP sequence reflects the post-relapse use of ISA-Kd and should not be interpreted as the cost of first-line VMP alone. Alternative second-line pathways were not explored in scenario analyses; consequently, the results should be interpreted as conditional on the treatment sequences specified in the model.

In conclusion, the results support a cautious interpretation. From a probabilistic perspective, Rd appears to be the preferred strategy at lower WTP thresholds, while D-VMP may become attractive only at substantially higher thresholds. DARA-Rd, despite its clinical advantages, does not achieve a favorable cost-effectiveness profile within the modeled horizon. Overall, these findings are consistent with the international literature in indicating that the main barrier to the economic sustainability of daratumumab-based frontline therapy lies not in its efficacy, but in its current acquisition cost (6, 43, 48). In this context, pricing policies and reimbursement strategies emerge as key determinants of cost-effectiveness rather than secondary considerations.

## Data Availability

The datasets presented in this article are not readily available because this study is based exclusively on simulated data and does not involve real patients. All simulated cohorts and the corresponding results are fully reproducible, having been generated using custom software developed in R (RStudio/Tidyverse 4.5.2), together with several supporting libraries (50). A complete specification of the input parameters, detailed descriptions of the algorithm used to estimate state utilities, and the full source code are available from the authors upon reasonable request. Requests to access the datasets should be directed to antonio.solimando@uniba.it.

## Footnotes

1 https://apps.automeris.io/wpd/.

2 https://zenodo.org/records/21408576. The deposited repository contains the complete R source code underlying the analysis, including the specification of all parameter values employed in the fixed base-case simulation and the full configuration of the prior distributions adopted for the probabilistic sensitivity analysis, thereby enabling exact reproduction of both the deterministic and the probabilistic results.
