# Supplementary material for: Integrating clinical and economic outcomes in the elderly myeloma management: a comparative cost-effectiveness analysis
Source: Front Oncol. 2026 Aug 24;16:1774263. doi: 10.3389/fonc.2026.1774263 (PMC13547032; doi:10.3389/fonc.2026.1774263)
Supplement: Supplementary file 1 [file DataSheet1.pdf]

Supplementary Material to: Integrating Clinical and Economic Outcomes in the Elderly Myeloma Management: A Comparative Cost-Effectiveness Analysis

1 Therapy scheduling

1.1 VMP

Patients receive 9 treatment cycles with VMP (1 cycle = 42 days = 6 weeks) according to the following dosing schedule:

- bortezomib: 1.3 mg/m^2 of body surface area (BSA) administered twice weekly during weeks 1, 2, 4, and 5 of the first cycle (for a total of 8 administrations), and once weekly during weeks 1, 2, 4, and 5 of cycles 2 to 9 (for a total of 4 administrations per cycle).
- melphalan: 9 mg/m^2 of BSA administered once daily on days 1-4 of each treatment cycle.
- prednisone: 60 mg/m^2 of BSA administered once daily on days 1-4 of each treatment cycle.

Up to cycle 9, patients who have neither died nor experienced disease progression remain in state [T]. Thereafter, the VMP regimen is discontinued, and the follow-up phase begins; consequently, from cycle 10 onwards, patients transition to state [M], and no further direct drug-related costs are incurred.

1.1.1 Second-line treatment: Isa-Kd

The therapeutic regimen consists of the following administrations:

- isatuximab: 10 mg/kg administered by infusion on days 1, 8, 16, and 22 of the first cycle (cycle duration: 28 days), and on days 1 and 15 from the second cycle onwards (4 administrations in cycle 1, 2 administrations per cycle thereafter).
- carfilzomib: administered by infusion on days 1, 2, 8, 9, 15, and 16 of the first 28-day cycle, at a dose of 20 mg/m^2 of BSA on days 1 and 2, and 56 mg/m^2 of BSA on the remaining days. From cycle 2 onwards, treatment is administered on days 1, 2, 8, 9, 15, and 16 of each cycle at a standard dose of 56 mg/m^2 of BSA (for a total of 6 administrations per cycle, with a slightly lower cumulative dose only in the first cycle).
- dexamethasone: 20 mg administered on days 1, 2, 8, 9, 15, and 16 of each 28-day cycle (6 administrations per cycle).

On days when isatuximab is administered, paracetamol and an antihistamine are also given at the same therapeutic dosage specified for D-VMP. Treatment is continued until disease progression or the occurrence of unacceptable toxicity.

1.2 D-VMP

The total duration of treatment consists of 9 cycles of 42 days each. Daratumumab is administered at a dose of 16 mg/kg according to the following schedule:

- cycle 1: once weekly on days 1, 8, 15, 22, 29, and 36 (for a total of 6 administrations in cycle 1).
- cycles 2-9: once weekly on days 1 and 22 (for a total of 2 administrations per cycle).
- cycle 9+: one administration every 4 weeks.

During cycles 1-9, the VMP regimen is administered in combination with daratumumab. The only modification is that the intravenous infusion of daratumumab is preceded by the administration of dexamethasone 20 mg, which replaces prednisone on day 1 of each VMP cycle, as well as paracetamol 650-1000 mg and chlorphenamine maleate (antihistamine) 20 mg.

Treatment is continued until disease progression or the occurrence of unacceptable toxicity. Accordingly, during cycles 1-9, patients who have neither died nor progressed remain in state [T], whereas from cycle 10 onwards they transition to state [M].

1.2.1 Second-line treatment: KRd

This regimen includes the following administrations:

- carfilzomib: administered by infusion on days 1, 2, 8, 9, 15, and 16 of a 28-day cycle, at 20 mg/m^2 of BSA on days 1 and 2 of cycle 1, and 27 mg/m^2 of BSA from day 8 of cycle 1 onwards. If BSA exceeds 2.2 m^2, the value 2.2 is used for dose calculation (6 administrations per cycle). For cycles 13 to 18, administration is limited to days 1, 2, 15, and 16 (4 administrations per cycle).
- lenalidomide: 25 mg administered on days 1--21 of each 28-day cycle (21 administrations per cycle).
- dexamethasone: 40 mg administered once daily on days 1, 8, 15, and 22 of each cycle (4 administrations per cycle).

From cycle 19 onwards, carfilzomib is discontinued, and treatment continues with the Rd regimen until further disease progression or unacceptable toxicity.

1.3 Rd

This regimen includes the following treatments:

- lenalidomide: 25 mg administered on days 1-21 of each 28-day cycle (21 administrations per cycle).
- dexamethasone: 40 mg administered once daily on days 1, 8, 15, and 22 of each cycle (4 administrations per cycle).

Treatment is continued indefinitely until documented disease progression or the occurrence of unacceptable toxicity. Under the Rd regimen, there is no distinct state [M], as patients remain on treatment until progression (and transition to second-line therapy) or death. The fact that the treatment cycle is 28 days is appropriately accounted for in the cost calculations, which are rescaled to a 42-day cycle.

1.3.1 Second-line treatment: Isa-Pd

This regimen includes the following treatments:

- isatuximab: 10 mg/kg administered by infusion on days 1, 8, 16, and 22 of the first cycle (cycle duration: 28 days), and on days 1 and 15 from the second cycle onwards (4 administrations in cycle 1, 2 administrations per cycle thereafter).
- pomalidomide: 4 mg administered on days 1--21 of each 28-day cycle (21 administrations per cycle).
- dexamethasone: 20 mg administered on days 1, 2, 8, 9, 15, and 16 of each 28-day cycle (6 administrations per cycle).

On days when isatuximab is administered, paracetamol and an antihistamine are also given at the same therapeutic dosage specified for D-VMP. Treatment is continued until disease progression or the occurrence of unacceptable toxicity.

1.4 DARA-Rd

In this treatment, each therapeutic cycle has a duration of 28 days. The treatments are administered according to the following schedule:

- daratumumab: the administration schedule differs from that adopted in D-VMP. Specifically:
  - cycles 1-2: 1800 mg administered by subcutaneous infusion on days 1, 8, 15, and 22 (4 administrations per cycle).
  - cycles 3-6: 1800 mg administered by subcutaneous infusion on days 1 and 15 (2 administrations per cycle).
  - cycles 7+: 1800 mg administered by subcutaneous infusion on day 1 (1 administration per cycle).
- lenalidomide: 25 mg administered on days 1-21 of each 28-day cycle (21 administrations per cycle).
- dexamethasone: administered according to the following schedule:
  - cycles 1-2: 20 mg once daily on days 1, 2, 8, 9, 15, 16, 22, and 23 (8 administrations per cycle).
  - cycles 3--6: 20 mg once daily on days 1, 2, 15, and 16 (4 administrations per cycle).
  - cycles 7+: 20 mg once daily on days 1 and 2 (2 administrations per cycle).

Treatment is continued until disease progression or the occurrence of unacceptable toxicity. The distinction between treatment and maintenance phases is more artificial than in the case of VMP (no therapy after 9 cycles) or D-VMP (one administration every 4 weeks after 9 cycles) but is retained for consistency across the treatment arms considered in the analysis.

1.4.1 Second-line treatment: VPd

Each therapeutic cycle has a duration of 21 days. The treatment schedule is as follows:

- bortezomib: administered according to the following regimen:
  - cycles 1-8: 1.3 mg/m^2 of BSA on days 1, 4, 8, and 11 of each cycle (4 administrations per cycle).
  - cycles 9+: 1.3 mg/m^2 of BSA on days 1 and 8 of each cycle (2 administrations per cycle).
- pomalidomide: 4 mg administered on days 1-14 of each cycle.
- dexamethasone: administered according to the following schedule:

- cycles 1-8: 20 mg once daily on days 1, 2, 4, 5, 8, 9, 11, and 12 (8 administrations per cycle).
- cycles 9+: 20 mg once daily on days 1, 2, 8, and 9 (4 administrations per cycle).

2 Cost table

A list of all costs mentioned in Subsection 2.4 as well as of main structural parameters is reported in the Table below:

**Demographic parameters (a)**	
Body weight	70.80 kg
Body height	170.70 cm
Body surface area (BSA)	1.82 m^2
**Costs per single administration (b)**	
daratumumab 1133 mg SC (2023/1st TM. updated price)	€2375.00
dexamethasone 20 mg IV (2023/03 updated price)	€4.79
dexamethasone 40 mg IV (2023/03 updated price)	€7.98
paracetamol 1000 mg IV (2023/03 updated price)	€5.29
chlorphenamine maleate 20 mg SC (2023/03 updated price)	€4.24
bortezomib 2.37 mg SC (2023/1st TM. updated price)	€28.07
melphalan 16.41 mg OS (2023/03 updated price)	€5.56
prednisone 60 mg OS (2023/03 updated price)	€1.76
carfilzomib 36.47 mg EV KRd cycle 1 (2023/1st TM. updated price)	€440.50
carfilzomib 49.24 mg EV (2023/1st TM. updated price)	€594.70
carfilzomib 102.12 mg (2023/1st TM. updated price)	€1234.00
lenalidomide 25 mg OS (2023/1st TM. updated price)	€1.20
isatumixab 708 mg EV (2023/1st TM. updated price)	€2159.00
lenalidomide 25 mg OS (2023/03 updated price)	€457.30
dexamethasone 40 mg IV LenDex (2023/03 updated price)	€7.98
**Other direct costs (c)**	
Day-hospital admissions (per year in 2008 Euros)	€499.90
Emergency rooms treatments (per year in 2008 Euros)	€83.50
General practitioner visit (per year in 2008 Euros)	€203.60
Specialist visit (per year in 2008 Euros)	€406.10
Clinical and diagnostic tests (per year in 2008 Euros)	€1406.00
Other drugs (c) (per year in 2008 Euros)	€685.90
Transfusions, radiotherapy (per year in 2008 Euros)	€25.70
Total (per year in 2008 Euros)	€3310.00
Total (per cycle in 2023/07 Euros)	€492.30
**Additional transition costs (d)**	
Infections	€5744.00
Hematologic complications	€3589.00
**Discount rate r (e)**	3%

For the correct interpretation of this table, the following points should be noted:

(a) The parameters reported were obtained from the literature. (1–3)
(b) To compute the total cost incurred for each drug within a treatment cycle, the cost per single administration should be multiplied by the number of administrations. For example, in the D-VMP regimen, there are 6 administrations of daratumumab during the first cycle of therapy at $t = 0$ (one per week). (4)
(c) Prices originally expressed in 2008 euros were updated to July 2023 values using the official conversion tool based on the Italian consumer price index for blue- and white-collar workers[1]). (5)
(d) This category includes drugs not administered during inpatient care, such as bisphosphonates (for osteolytic lesions), antibiotics, antivirals, antianemic agents, allopurinol, analgesics, antihemorrhagics, antihistamines, antihypertensives, antiulcer agents, fluconazole, phenobarbital, psychotropic drugs, statins, and vitamin D3. (6)
(e) See: (7)

The complete set of transition probabilities, utility values, and probability distributions (together with their associated parameters) used in the probabilistic simulations can be directly derived from the simulation code. All simulated cohorts and the corresponding results are fully reproducible, having been generated using custom software developed in R (RStudio/Tidyverse 4.5.2), together with several supporting libraries. A complete specification of the input parameters, a detailed description of the algorithm used to estimate state utilities, and the full source code are available in the Zenodo repository referenced in the manuscript.

Additional References

1. Burton RF. Estimating body surface area from mass and height: Theory and the formula of Du Bois and Du Bois. *Ann Hum Biol* (2008) 35:170–184. doi: 10.1080/03014460801908439

2. Walpole SC, Prieto-Merino D, Edwards P, Cleland J, Stevens G, Roberts I. The weight of nations: an estimation of adult human biomass. *BMC Public Health* (2012) 12:439. doi: 10.1186/1471-2458-12-439

3. Roser M, Appel C, Ritchie H. Human Height. *Our World Data* (2013) https://ourworldindata.org/human-height [Accessed September 16, 2023]

4. AIFA. AIFA Italian Medicine Agency: Pricing and Rembursement. (2025) https://www.aifa.gov.it/en/prezzi-e-rimborso

5. Petrucci MT, Calabrese E, Levi A, Federico V, Ceccolini M, Rizzi R, Gozzetti A, Falco P, Lazzaro C, Martelli E, et al. Cost of Illness in Patients with Multiple Myeloma in Italy: The CoMiM Study. *Tumori J* (2013) 99:e193--e202. doi: 10.1177/030089161309900434

6. Ministero della Salute. Principali caratteristiche dei Diagnosis Related Groups (DRG). (2023) https://tinyurl.com/bdd8wpu2

[1] https://rivaluta.istat.it/

7. Eurostat. Eurostat Statistics Explained – Inflation in the Euro Area. (2023) https://tinyurl.com/yck9hxak
