# Supplementary material for: Integrating clinical and economic outcomes in the elderly myeloma management: a comparative cost-effectiveness analysis
Source: Front Oncol. 2026 Aug 24;16:1774263. doi: 10.3389/fonc.2026.1774263 (PMC13547032; doi:10.3389/fonc.2026.1774263)
Supplement: Supplementary file 2 [file DataSheet2.pdf]

ISPOR

Improving healthcare decisions

Consolidated Health Economic Evaluation Reporting Standards (CHEERS) 2022 Checklist

The CHEERS 2022 statement replaces the 2013 CHEERS statement, which should no longer be used. The CHEERS 2022 checklist contains 28 items with accompanying descriptions. Checklist users should indicate the section of the manuscript where relevant information can be found. The authors recommend using a section heading with a paragraph number. If an item does not apply to a particular economic evaluation, checklist users are encouraged to report "Not Applicable." If information is otherwise not reported, checklist users are encouraged to write, "Not Reported." Users should avoid the term "Not Conducted" as CHEERS is intended to guide and capture reporting. Additional information on CHEERS 2022 can be found here.

Title

1. Title

Identify the study as an economic evaluation and specify the interventions being compared.

The title identifies the study as a comparative cost-effectiveness analysis. The four compared first-line interventions (VMP, D-VMP, Rd, DARA-Rd) are not named in the title itself but are specified in the Abstract and Introduction.

Abstract

2. Abstract

Provide a structured summary that highlights context, key methods, results, and alternative analyses.

Abstract. A structured abstract (Introduction, Methods, Results, Conclusion) summarises context (elderly transplant-ineligible MM), methods (state-transition Markov model parameterised on ALCYONE and MAIA, INHS perspective, PSA with CEACs), main results (ICER of D-VMP vs Rd ≈ €160,000; DARA-Rd and VMP dominated) and alternative analyses through the probabilistic sensitivity analysis and WTP-threshold interpretation.

Introduction

3. Introduction: Background and Objectives

Give the context for the study, the study question, and its practical relevance for decision making in policy or practice.

Introduction. The background provides epidemiological context (GLOBOCAN: ≈176,000 new cases/year worldwide, >60% aged ≥65; ≈5,000 new Italian cases/year, median age >70) and identifies the evidence gap, namely the lack of simultaneous multi-arm cost-effectiveness analyses. Policy relevance for the Italian setting (AIFA HTA process, ICER as a reimbursement criterion, regional price heterogeneity) is described. The stated objective is to assess the cost-effectiveness of four first-line regimens in elderly or transplant-ineligible MM patients.

Methods

4. Health economic analysis plan

Indicate whether a health economic analysis plan was developed and where available.

Not Reported. The manuscript does not reference a separately developed or publicly available pre-specified health economic analysis plan; the analytic approach is described within the Methods section and the Supplementary Material.

---

5. Study population

Describe characteristics of the study population (such as age range, demographics, socioeconomic, or clinical characteristics).

Methods (Material and Methods, opening paragraph). The simulated cohort comprises patients aged ≥70 years, or younger patients with comorbidities precluding ASCT, newly diagnosed with MM according to EHA–ESMO Clinical Practice Guidelines. Baseline characteristics are derived from the pivotal trials: ALCYONE (n=706; median age 71.0 years, range 40–93) for VMP/D-VMP and MAIA (n=736; median age 73.0 years, range 50–90) for Rd/DARA-Rd.

---

6. Setting and location

Provide relevant contextual information that may influence findings.

Methods (Perspective) and Introduction. The study is set within the Italian National Health Service (INHS). Unit costs reflect prices negotiated in 2023 by the Hospital Consorziale Policlinico in Bari, Apulia, with prices noted as

variable across Regions and hospital consortia. The Introduction details the Italian institutional context (AIFA-governed HTA and pricing, regional negotiation, aging population).

7. Comparators

Describe the interventions or strategies being compared and why chosen.

Methods and Introduction. Four first-line strategies for transplant-ineligible MM are compared: VMP, D-VMP, Rd and DARA-Rd, selected as the principal guideline-relevant first-line options. Each is evaluated as a pre-specified treatment sequence (VMP→ISA-Kd, Rd→ISA-Pd, D-VMP→KRd, DARA-Rd→VPd), with the rationale for each second-line choice justified per EHA–ESMO guidelines (mechanism of action, response duration, avoidance of cross-resistance).

8. Perspective

State the perspective(s) adopted by the study and why chosen.

Methods (Perspective). Costs are evaluated from the perspective of the Italian public payer (INHS), including drug and medical-supply consumption, laboratory services, equipment and professional fees; patient out-of-pocket expenses are excluded as negligible. The perspective is justified by the centralised AIFA reimbursement governance of the INHS.

9. Time horizon

State the time horizon for the study and why appropriate.

Methods. The simulation horizon is 45 cycles of 42 days (≈5.25 years). It is justified as consistent with the median overall survival reported in the literature and with the follow-up of the ALCYONE and MAIA trials, deliberately kept finite to limit long-term extrapolation uncertainty.

10. Discount rate

Report the discount rate(s) and reason chosen.

Methods (Costs and utilities / Perspective). In the fixed-input base case, both future costs and QALYs are discounted at an annual rate of 3%, in accordance with Italian pharmacoeconomic recommendations. The same rate is applied to costs and outcomes in the absence of evidence supporting differential discounting; alternative rates (1.5–7.5%) are explored in the PSA.

© 2022 ISPOR — The Professional Society for Health Economics and Outcomes Research
International Society for Pharmacoeconomics and Outcomes Research, Inc

11. Selection of outcomes

Describe what outcomes were used as the measure(s) of benefit(s) and harm(s).

Methods. The primary outcome is cost-effectiveness expressed as the incremental cost-effectiveness ratio (ICER, €/QALY), with health benefit measured in QALYs. Secondary endpoints are PFS, OS and the incidence of treatment-related adverse events; severe infections [I] and hematologic complications [H] are captured as harms via transient one-cycle states.

---

12. Measurement of outcomes

Describe how outcomes used to capture benefit(s) and harm(s) were measured.

Methods (Disease relapse and death; Transition probabilities). PFS and OS were reconstructed from digitized Kaplan–Meier curves of ALCYONE and MAIA (WebPlotDigitizer) and fitted with parametric survival models (exponential vs Weibull, selected by BIC) to derive cycle-specific transition probabilities. QALYs were computed by weighting health-state utilities by the time spent in each state per cycle.

---

13. Valuation of outcomes

Describe the population and methods used to measure and value outcomes.

Methods (Setting Utilities). Health-state utilities for [T], [M] and [R] were obtained from EQ-5D-5L data of the same trial cohorts (ALCYONE for VMP/D-VMP; MAIA for Rd/DARA-Rd). Incremental disutilities for [I] and [R] were sourced from an EQ-5D-5L dataset in a relapsed/refractory DLBCL cohort (>60% aged ≥65), and the disutility for [H] from an EORTC QLQ-C30-based estimate of febrile neutropenia in NSCLC. Utilities are defined on a 0–1 cardinal scale.

---

14. Measurement and valuation of resources and costs

Describe how costs were valued.

Methods (Perspective; Costs and utilities). Direct medical costs (drug acquisition and administration, adverse-event management for infections and hematologic toxicities, and second-line therapy after relapse) were valued from the INHS perspective. Unit costs are reported in the companion paper, based on 2023 prices negotiated by the Policlinico di Bari; full unit costs and treatment-cycle cost calculations are provided in Supplementary Material, Section 2.

© 2022 ISPOR — The Professional Society for Health Economics and Outcomes Research

International Society for Pharmacoeconomics and Outcomes Research, Inc

15. Currency, price date, and conversion

Report the dates of the estimated resource quantities and unit costs, plus the currency and year of conversion.

Methods (Perspective). All costs are expressed in euros (€) for the reference year 2023, based on 2023 negotiated prices. No currency conversion was required, as the analysis is single-currency and single-country (Italy).

---

16. Rationale and description of model

If modeling is used, describe in detail and why used. Report if the model is publicly available and where it can be accessed.

Methods (Model States; Transition probabilities) and Figure 1. A non-stationary discrete-time cohort state-transition (Markov) model with six mutually exclusive states ([T], [I], [H], [M], [R], [D]) and a time-varying transition matrix Pt was used. The cohort approach is justified as the standard formalism for cohort-level cost-effectiveness analysis. The model structure is depicted graphically in Figure 1 across three simulation phases. The source code was developed in R and is available is available at the Zenodo repository: https://zenodo.org/records/21408576

---

17. Analytics and assumptions

Describe any methods for analyzing or statistically transforming data, any extrapolation methods, and approaches for validating any model used.

Methods (Transition probabilities; Internal and external consistency). Analytic methods include Kaplan–Meier reconstruction with inversion algorithms, parametric survival extrapolation (exponential/Weibull by BIC), residual computation of the maintenance-state probability as the complement to one, and transient one-cycle adverse-event states. Internal validation of structural and computational consistency was performed; external validation was not undertaken because no suitable independent dataset reproduced the full set of strategies, states and follow-up. PSA is interpreted as an uncertainty analysis, not a formal validation.

---

18. Characterizing heterogeneity

Describe any methods used for estimating how the results of the study vary for subgroups.

Not Reported. No subgroup or heterogeneity analysis of results was conducted. Patient frailty, a recognised modifier of tolerability and outcomes, was not explicitly modeled; this is acknowledged as a limitation in the Discussion and identified as a direction for future research.

© 2022 ISPOR — The Professional Society for Health Economics and Outcomes Research
International Society for Pharmacoeconomics and Outcomes Research, Inc

19. Characterizing distributional effects

Describe how impacts are distributed across different individuals or adjustments made to reflect priority populations.

Not Applicable. The analysis is a cohort-level model that does not assess the distribution of impacts across individuals or priority populations, and no equity weighting or distributional adjustment was applied.

20. Characterizing uncertainty

Describe methods to characterize any sources of uncertainty in the analysis.

Methods (Probabilistic sensitivity analysis). Uncertainty was characterised through a PSA with N=50,000 sampled parameter sets: cost components modeled with Gamma distributions (method of moments), negotiated drug prices with Gaussian priors ($\sigma=0.23\mu$, i.e. ±20% at $\mu\pm3\sigma$), discount rates with a discrete uniform distribution (1.5–7.5%), and transition probabilities and utilities sampled from suitable distributions. Results are summarised via cost-effectiveness acceptability curves (CEACs).

21. Approach to engagement with patients and others affected by the study

Describe any approaches to engage patients or service recipients, the general public, communities, or stakeholders (eg, clinicians or payers) in the design of the study.

Not Applicable. The study is a model-based simulation using published and secondary data with no human participants; no patient, public or stakeholder engagement was undertaken in its design.

Results

22. Study parameters

Report all analytic inputs (eg, values, ranges, references) including uncertainty or distributional assumptions.

Methods and Supplementary Material (Section 2). All analytic inputs are reported with their sources and uncertainty assumptions: transition probabilities derived from ALCYONE and MAIA; health-state utilities and disutilities from the cited literature; unit costs from the companion paper. Distributional assumptions for the PSA (Gamma, Gaussian, discrete uniform) are specified, with the full parameter set detailed in the Supplementary Material.

23. Summary of main results

© 2022 ISPOR — The Professional Society for Health Economics and Outcomes Research

International Society for Pharmacoeconomics and Outcomes Research, Inc

Report the mean values for the main categories of costs and outcomes of interest and summarize them in the most appropriate overall measure.

Results (Table 1). Mean total costs and QALYs per strategy: Rd €60,483 / 1.53 QALY (ND); D-VMP €88,331 / 1.70 QALY (incremental €27,848 and 0.17 QALY vs Rd; ICER €160,804/QALY; ND); DARA-Rd €133,770 / 1.70 QALY (dominated); VMP €203,006 / 1.58 QALY (dominated). The ICER is used as the overall summary measure within an extended-dominance framework.

---

24. Effect of uncertainty

Describe how uncertainty about analytic judgments, inputs, or projections affects findings. Report the effect of choice of discount rate and time horizon, if applicable.

Results (Results from the PSA) and Discussion. CEACs indicate that Rd has the highest probability of being cost-effective at WTP thresholds below €250,000/QALY, while D-VMP becomes the preferred strategy above that level. Incorporating parameter variability through the PSA shifts the threshold at which D-VMP becomes most likely cost-effective upward (from ≈€160,000 in the base case to ≈€250,000). The discount rate was varied within the PSA, and the influence of the finite, trial-anchored time horizon on ICERs and QALY differences is discussed.

---

25. Effect of engagement with patients and others affected by the study

Report on any difference patient/service recipient, general public, community, or stakeholder involvement made to the approach or findings of the study.

Not Applicable. As no patient, public or stakeholder engagement was conducted, no resulting effect on the study approach or findings is reported.

Discussion

26. Study findings, limitations, generalizability, and current knowledge

Report key findings, limitations, ethical, or equity considerations not captured and how these could impact patients, policy, or practice.

Discussion and Conclusions. Key findings (Rd preferred at lower WTP; D-VMP attractive only at high WTP; DARA-Rd clinically superior yet not cost-effective at current pricing) are situated within the international literature. Limitations are detailed: sensitivity of Kaplan–Meier reconstruction and parametric extrapolation, a single fixed second-line per arm, frailty not modeled, the constant-hazard exponential OS assumption, reliance on two

separate trials with no head-to-head or formal indirect comparison, and between-trial heterogeneity. Generalizability to health systems with analogous structural features is discussed; ethical review is addressed in the Ethics Statement.

Other Relevant Information

27. Source of funding

Describe how the study was funded and any role of the funder in the identification, design, conduct, and reporting of the analysis.

Funding. The study was supported by the 2023 SIMI Research Award (CAMEL, to AGS), the INNOVA network (Ministero della Salute, PNC-E3-2022-23683266), and the Italian Complementary National Plan PNC-I.1 project DARE. The funding sources are listed; an explicit statement on the role of the funders in the design, conduct or reporting of the analysis is not provided and could be added.

---

28. Conflicts of interest

Report authors' conflicts of interest according to journal or International Committee of Medical Journal Editors requirements.

Conflict of Interest. The section discloses speaker honoraria and advisory-board roles for several authors (AV, AGS, RR) with multiple pharmaceutical companies, stating that these did not influence the data or opinions reported. The disclosure is consistent with ICMJE-style reporting requirements.
